# Exosomes derived from calcium oxalate-treated macrophages promote apoptosis of HK-2 cells by promoting autophagy

**DOI:** 10.1080/21655979.2021.2012622

**Published:** 2022-01-17

**Authors:** Lei Yan, Jinhu Chen, Weihua Fang

**Affiliations:** a Department of Urology, The First Affiliated Hospital of Anhui Medical University, Hefei, China; b Institute of Urology, the First Affiliated Hospital of Anhui Medical University, Hefei, China

**Keywords:** Calcium oxalate, macrophage, exosomes, apoptosis, autophagy, kidney stone

## Abstract

Calcium oxalate (CaOx) crystals are the main component of kidney stones. Macrophages have the function of eliminating these crystals, and the underlying mechanism remains unclear. Here, we attempted to determine the role of macrophage-derived exosomes exposed to CaOx crystals in regulating apoptosis of human proximal tubular cells (HK-2). Exosomes (CaOx-Exo) were isolated from CaOx-treated macrophages and then incubated with HK-2 cells. CaOx-Exo treatment reduced cell viability and promoted apoptosis of HK-2 cells. The expression of Caspase-3 and Bax was increased, and Bcl-2 expression was decreased in HK-2 cells following CaOx-Exo treatment. Moreover, CaOx-Exo treatment caused an increase of LC3-II/LC3-I ratio and Beclin-1 expression and a downregulation of p62 in HK-2 cells. GFP-LC3 puncta were increased in HK-2 cells following CaOx-Exo treatment. Additionally, CaOx-Exo-treated HK-2 cells were treated with 3-methyladenine (3-MA) to inhibit autophagy activity. 3-MA treatment weakened the impact of CaOx-Exo on cell viability and apoptosis of HK-2 cells. 3-MA treatment also reduced the LC3-II/LC3-I ratio and Beclin-1 expression and enhanced p62 expression in CaOx-Exo-treated HK-2 cells. In conclusion, these data demonstrated that exosomes derived from CaOx-treated macrophages promote apoptosis of HK-2 cells by promoting autophagy. Thus, this work suggests that macrophage-derived exosomes may play a vital role in CaOx-induced human proximal tubular cell damage.

## Introduction

Kidney stones are one of the most common diseases in urology. The prevalence and incidence of kidney stones are on the rise [1]. Kidney stones are related to various factors such as genetics, environment and metabolism [2]. The main component of kidney stones is calcium oxalate (CaOx) [3]. The recurrence rate of CaOx kidney stones after cure is as high as 60% to 80%, which seriously affects life and health of patients. Renal tubular epithelial cells are the key cells for the formation of stones. The supersaturated oxalic acid and calcium in urine cause damage to renal tubular epithelial cells, provide effective sites for the adhesion of CaOx crystals, and promote the aggregation and growth of crystals [4]. On the other hand, cell damage can cause a series of disorders that regulate the process of crystal formation, and ultimately lead to the formation of CaOx kidney stones [5]. Therefore, studying the pathogenesis of CaOx kidney stones and its prevention and treatment strategies has always been one of the important topics in the clinical research in urology.

Autophagy is a life phenomenon widely existing in eukaryotic cells [6]. It is a cellular mechanism that maintains intracellular balance by degrading proteins and organelles [7]. Previous studies have reported that autophagy is involved in the regulation of renal tubular epithelial cell damage and kidney stone formation caused by CaOx crystals [8–11]. CaOx crystals induce autophagy activation in renal tubular epithelial cells, and inhibition of autophagy significantly improves the CaOx crystal-induced renal tubular epithelial cell apoptosis and renal CaOx crystal deposition [12].

Macrophages are important inflammatory cells with phagocytic function in the organism. The expression of macrophage-related genes is significantly higher in renal papillary Randall plaques of kidney stone patients than that in non-Randall plaque tissues [13]. Under the stimulation of oxalic acid or CaOx crystals, kidney cells produce various inflammatory factors and induce monocytes or macrophages to migrate to the place where the stone crystals are deposited. Monocytes or macrophages can phagocytize and transport crystals and further promote the occurrence and development of inflammation [14–16]. Therefore, macrophages are closely related to the formation of kidney stones. Exosomes are biologically active vesicles secreted by cells with a diameter of about 30–150 nm. Exosomes play an important role in the communication between macrophages and other cells [17]. Nilubon et al. have found that CaOx-treated macrophages promote the expression of IL-8 in renal tubular epithelial cells by secreting exosomes and promote the migration of neutrophils, thereby regulating the inflammatory response of kidney stones [18].

In this work, we speculated whether exosomes derived from macrophages can regulate renal tubular epithelial cell damage by regulating autophagy. Thus, the aim of this study was to investigate the biological role of CaOx-treated macrophage-derived exosomes in kidney stone development. This work confirmed that exosomes derived from CaOx crystal-treated macrophages promoted apoptosis of proximal tubular cells by promoting autophagy.

## Materials and methods

### Cell culture and treatment

Human proximal tubular HK-2 cells (ATCC, Manassas, VA, USA) and THP-1 monocytes (ATCC) were cultured in RPMI-1640 medium (Gibco; Grand Island, NY) in the presence of 10% fetal bovine serum (FBS, Gibco) and 100 U/mL penicillin/streptomycin (Solarbio, Beijing, China) at 37°C and 5% CO_2_. For macrophage induction, THP-1 monocytes were incubated with 100 ng/mL phorbol ester (PMA, Sigma-Aldrich, St. Louis, MO, USA) for 48 h as previously described [19]. The cells were washed with precooled phosphate buffer saline (PBS) to collect the adherent THP-1 macrophages.

CaOx crystals (CaC_2_O_4_; purity: ≥ 99.9%; density: 2.2 g/mL at 25°C (lit.); Sigma-Aldrich) (100 mg) were dissolved in sterile water (100 mL) and sonicated to obtain a suspension of CaOx crystals (1 mg/mL). The suspension of CaOx crystals was stored at 4°C, and mixed well before use. THP-1 macrophages were treated with 100 μg/mL CaOx crystals or PBS for 24 h as previously reported [20]. Exosomes were separated from the THP-1 macrophages following the treatment of CaOx crystals or PBS (CaOx-Exo or PBS-Exo). HK-2 cells were incubated with 30 μg CaOx-Exo, PBS-Exo or combined with 5 mM 3-methyladenine (3-MA; Sigma-Aldrich) for 24 h.

### Isolation and identification of exosomes

Isolation and identification of exosomes were carried out as previously reported [21]. Exosomes were isolated from the CaOx or PBS-treated THP-1 macrophages using a TransExo^TM^ Cell Media Exosome Kit (Transgen, Beijing, China) as the protocol described. Briefly, cell supernatant was centrifuged at 3000 × *g*, 4°C for 30 min to remove the residual cells and debris. Then, cell supernatant was incubated with exosome precipitation solution at 4°C overnight. The precipitated exosomes were collected by centrifugation at 10000 × *g,* 4°C for 30 min. Finally, the exosomes were resuspended in exosome resuspension solution and stored at −80°C for further use. The ultrastructure and particle size of CaOx-Exo or PBS-Exo were analyzed using Transmission electron microscopy (Thermo Fisher Scientific, Waltham, MA, USA) and NanoSight nanoparticle tracking analysis (NanoSight, Salisbury, UK). The expression of exosome markers, CD9 and CD63, in CaOx-Exo or PBS-Exo was examined by performing Western blot (WB).

### Cell viability

Cell viability of HK-2 cells was assessed by CCK-8 assay using Cell Counting Kit-8 (Beyotime Biotechnology, Shanghai, China) as previously reported [22]. Briefly, HK-2 cells were incubated with CCK-8 reagent at 37°C for 1 h. The optical density was detected at 450 nm using a microplate reader (Thermo Fisher Scientific).

### Apoptosis

Apoptosis of HK-2 cells was assessed by flow cytometry using a Annexin V-FITC Apoptosis Detection Kit (Beyotime Biotechnology) following as previously reported [22]. In brief, after CaOx-Exo or 3-MA treatment, HK-2 cells were stained with 5 μL of Annexin V-FITC and 10 μL of propidium iodide at 25°C in the dark for 20 min. The apoptotic cells were analyzed on a Beckman Coulter flow cytometer (Becton Dickinson, New Jersey, USA) within 1 h.

### Western blot (WB)

Western blot was performed as previously described [23]. Total protein was extracted from exosomes or HK-2 cells or using an Exosomes Protein Extraction Kit (BestBio, Beijing, China) or radioimmunoprecipitation assay buffer (Sigma-Aldrich). Protein samples were (20 μg proteins each sample) were resolved by 10% sodium dodecyl sulfate polyacrylamide gel electrophoresis, and the resolved proteins were then transferred onto a polyvinylidene fluoride membrane (Whatman, Dassel, Germany). The membranes were treated with 5% skim milk to block the nonspecific bindings and then incubated with the primary antibodies, CD63 (1:1000), CD9 (1:1000), Caspase-3 (1:1000), Bax (1:1000), Bcl-2 (1:50) LC3A/LC3B (1:1000), Beclin-1 (1:1000) or GADPH (1:2000) at 4°C overnight. The membranes were incubated with HRP-conjugated secondary antibodies (1:10000) at 25°C for 1 h. All the primary and secondary antibodies were obtained from Thermo Fisher Scientific. The immunoreactive bands were developed by electrochemiluminescence reagent (Pierce, Minneapolis, USA) and then analyzed by Image J software.

### Detection of GFP-LC3 puncta

GFP-LC3 puncta was detected following the protocols of the previous study [23]. HK-2 cells were seeded into 6-well plates at a concentration of 1 × 10^4^ cells per well and then infected with 2 µL of GFP-LC3 adenovirus vector (Ad-GFP-LC3) (Sciben, Nanjing, China). After 24 h of infection, cells were collected, washed with PBS, then fixed with 4% paraformaldehyde and mounted with a fluorescent mounting medium (Dako, Agilent Technologies, Santa Clara, CA, USA). The green fluorescent spots were observed under a fluorescence microscope (Leica, Wetzlar, Germany).

### Statistical analysis

Each assay was performed for 3 times, and the results were presented as mean ± standard deviation. GraphPad Prism 7 (GraphPad Software, Inc., La Jolla, CA, USA) was used for statistical analysis. The statistical differences between groups were analyzed applying Two-tailed Student’s *t*-test or two-way analysis of variance. A p-value lower than 0.05 (*P* < 0.05) was considered as a significant difference.

## Results

Kidney stone is one of the most common diseases in urology. The aim of this study was to investigate whether exosomes derived from CaOx crystal-treated macrophages can promote apoptosis of proximal tubular cells by promoting autophagy. First, macrophages were treated with CaOx crystals to mimic *in vivo* conditions within the kidney, and exosomes were isolated from the CaOx crystal-treated macrophages. Then, we explored the impact of CaOx-Exo on cell viability, apoptosis and autophagy of HK-2 cells. The data showed that exosomes derived from CaOx crystal-treated macrophages promoted apoptosis of proximal tubular cells by promoting autophagy.

### Identification of exosomes

**Figure 1.**
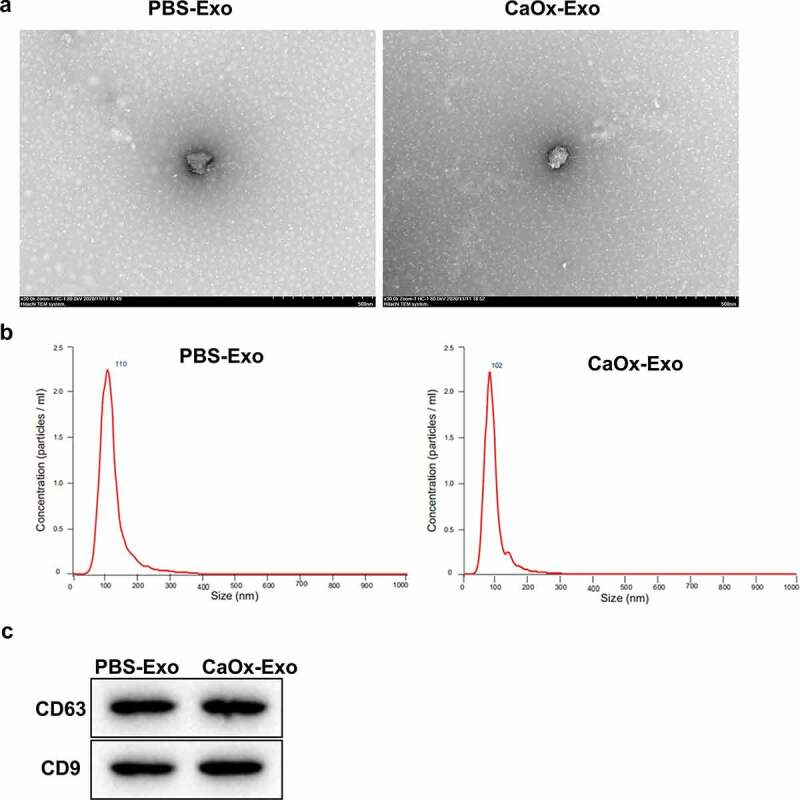
Identification of exosomes. The ultrastructure (a) and particle size (b) of exosomes isolated from the CaOx or PBS-treated THP-1 macrophages. Scar bar = 500 nm. (c) WB was used to confirm the expression of CD63 and CD9 in exosomes of CaOx or PBS-treated THP-1 macrophages.

We separated exosomes from the CaOx or PBS-treated THP-1 macrophages and examined the ultrastructure and particle size of exosomes. As shown in Figure 1(a,b), CaOx-Exo and PBS-Exo exhibited a typical spherical shape with a size of approximately 100 nm. WB was performed to assess the expression of exosome markers (CD9 and CD63) in exosomes, showing that CD9 and CD63 were highly expressed in CaOx-Exo and PBS-Exo Figure 1(c). Thus, the exosomes were successfully separated from the macrophages, and can be used for further analysis.

### CaOx-Exo treatment repressed cell viability and promoted apoptosis of HK-2 cells

**Figure 2.**
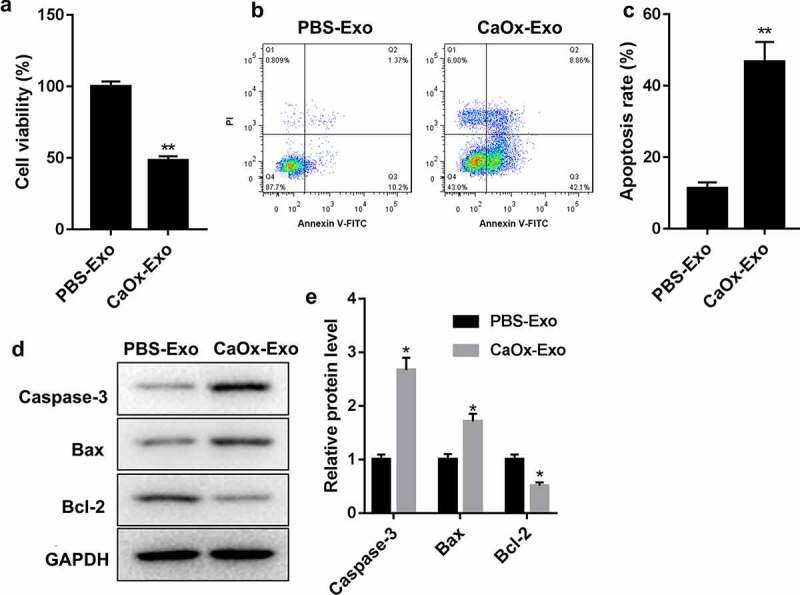
CaOx-Exo repressed cell viability and promoted apoptosis in HK-2 cells. (a) CCK-8 assay was performed to assess cell viability of HK-2 cells following treatment of CaOx-Exo or PBS-Exo. (b-c) Flow cytometry was performed to detect apoptosis of HK-2 cells following treatment of CaOx-Exo or PBS-Exo. (d-e) The expression of Caspase-3, Bax and Bcl-2 in HK-2 cells in the presence of CaOx-Exo or PBS-Exo was examined by WB. *P < 0.05, **P < 0.01 vs. PBS-Exo.

The impact of CaOx-Exo on cell viability and apoptosis of HK-2 cells was detected by CCK-8 assay and flow cytometry. As shown in Figure 2a, cell viability was severely decreased in HK-2 cells following CaOx-Exo treatment. The data obtained from flow cytometry revealed that CaOx-Exo treatment significantly enhanced the apoptotic cells in HK-2 cells Figure 2(b,c). We also found that the expression of pro-apoptotic protein Caspase-3 and Bax was increased in CaOx-Exo-treated HK-2 cells. Compared with PBS-Exo treatment, CaOx-Exo treatment notably repressed the expression of anti-apoptotic protein Bcl-2 in HK-2 cells Figure 2(d,e). Thus, these data showed that CaOx-Exo treatment repressed cell viability and promoted apoptosis of HK-2 cells.

### CaOx-Exo treatment promoted autophagy of HK-2 cells

**Figure 3.**
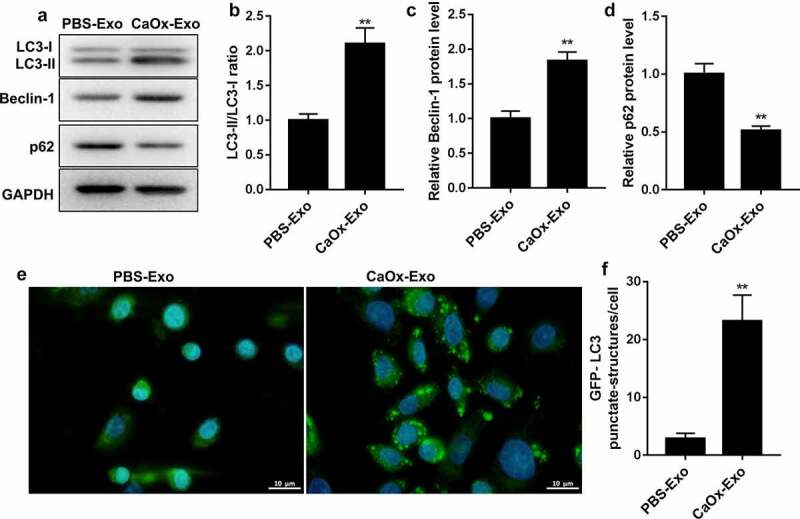
CaOx-Exo promoted autophagy of HK-2 cells. HK-2 cells were treated with CaOx-Exo or PBS-Exo. (a-d) The expression of LC3-I, LC3-II, Beclin-1 and p62 in HK-2 cells was measured by WB. (e-f) GFP-LC3 punctate was assessed. Scar bar = 10 μm. **P < 0.01 vs. PBS-Exo.

The influence of CaOx-Exo on autophagy of HK-2 cells was verified by WB and GFP-LC3 punctate. The results of WB revealed that the expression of LC3-II/LC3-I ratio and Beclin-1 were notably increased in HK-2 cells in the presence of CaOx-Exo Figure 3(a-c). However, the protein expression of p62 was severely decreased in HK-2 cells following CaOx-Exo treatment Figure 3(a-d). Moreover, compared with PBS-Exo treatment, CaOx-Exo treatment notably increased the GFP-LC3 puncta in HK-2 cells Figure 3(e,f). Therefore, CaOx-Exo promoted autophagy of HK-2 cells.

### CaOx-Exo promoted apoptosis of HK-2 cells by promoting autophagy

**Figure 4.**
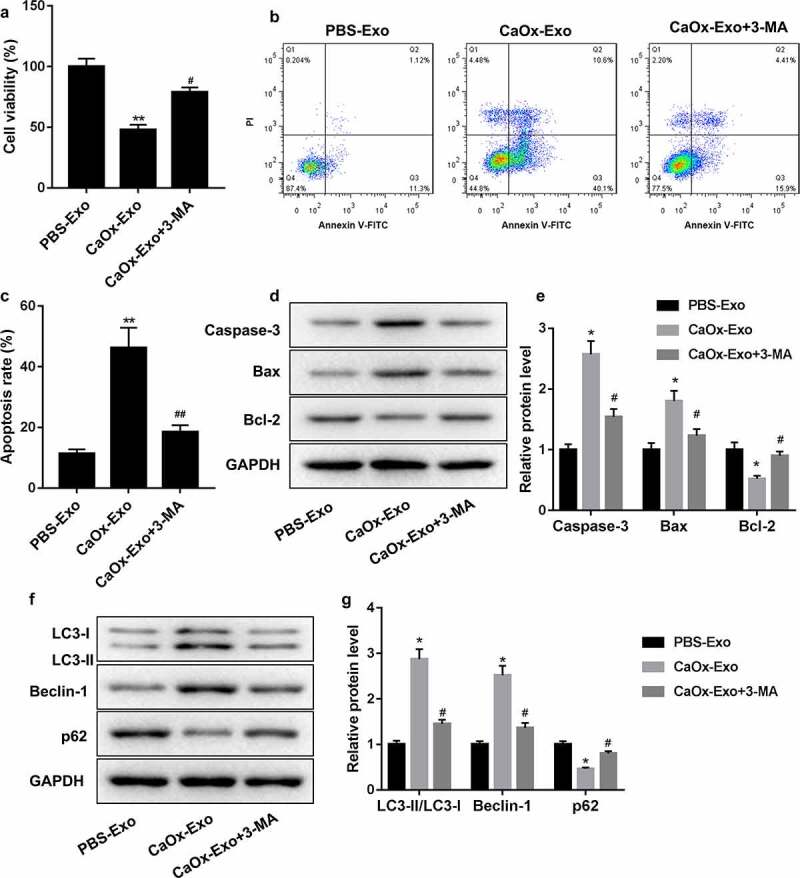
CaOx-Exo promoted apoptosis of HK-2 cells by promoting autophagy. HK-2 cells were treated with CaOx-Exo, PBS-Exo or combined with 3-MA. (a) CCK-8 assay was performed to assess cell viability of HK-2 cells. (b-c) HK-2 cell apoptosis was detected by flow cytometry. (d-g) The expression of Caspase-3, Bax, Bcl-2, LC3-I, LC3-II, Beclin-1 and p62 in HK-2 cells was examined by WB. *P < 0.05, **P < 0.01 vs. PBS-Exo; ^#^*P* < 0.05, ^##^*P* < 0.01 vs. CaOx-Exo.

Finally, we determined whether CaOx-Exo can affect apoptosis of HK-2 cells by regulating autophagy. CaOx-Exo-treated HK-2 cells were treated with 3-MA to inhibit autophagy activity. CaOx-Exo treatment repressed cell viability of HK-2 cells, which was effectively abrogated by 3-MA treatment Figure 4(a). Flow cytometry results uncovered that CaOx-Exo treatment led to a boost in apoptosis of HK-2 cells. 3-MA treatment weakened the CaOx-Exo-induced apoptosis of HK-2 cells Figure 4(b,c). Furthermore, WB data showed that CaOx-Exo treatment caused an upregulation of Caspase-3 and Bax and a downregulation of Bcl-2 in HK-2 cells. CaOx-Exo treatment also enhanced the LC3-II/LC3-I ratio and Beclin-1 expression and repressed p62 expression in HK-2 cells. The influence conferred by CaOx-Exo on these apoptosis and autophagy-related proteins was abolished by 3-MA treatment Figure 4(d-g). Taken together, these findings confirmed that CaOx-Exo promoted apoptosis of HK-2 cells by promoting autophagy.

## Discussion

In recent years, studies have found that a large number of macrophages infiltrate in the kidney with stones [24,25]. M2 macrophages protect HK-2 cells from oxidative stress and apoptosis by regulating p38 MAPK and Akt signaling pathways, thereby reducing the formation of kidney stones [26]. Macrophages phagocytize crystal deposits by releasing various cytokines and complement components that are essential for the inflammatory immune response [27]. Exosomes derived from CaOx monohydrate-treated macrophages increase the levels of IL-8 in renal tubular cells and promote the migration of neutrophil, indicating that macrophages take part in CaOx monohydrate-induced inflammatory response through the exosome pathway [28]. A previous study has confirmed that ursolic acid alleviates CaOx crystal-induced cell apoptosis, oxidative damage and inflammatory response in kidney stone rats and HK-2 cells [29]. This work explored whether CaOx-Exo can affect the development of kidney stone through regulating cell apoptosis and autophagy. We found that CaOx-Exo reduced cell viability and enhanced apoptosis and autophagy of HK-2 cells.

Both autophagy and apoptosis are programmed cell death. Autophagy is a series of biochemical processes in which eukaryotic cells ‘self-digest’ by degrading their own cytoplasm and organelles [30]. It is significantly different from apoptosis in biochemical metabolic pathways and morphology. However, autophagy and apoptosis can coordinate transformation through internal molecular regulation mechanisms to jointly promote cell death [31]. In kidney stone mouse models, the levels of cell apoptosis, autophagy-endoplasmic reticulum stress and crystal deposition are significantly increased, which is abolished by atorvastatin [9]. Autophagy is activated in the ethylene glycol-induced rat model of CaOx nephrolithiasis, and the levels of apoptosis, crystal deposition and renal injury are also increased [12]. Inhibition of endoplasmic reticulum stress-induced autophagy can repress cell damage and apoptosis through PERK-eIF2α signaling pathway, and thus represses the formation of kidney stones [32]. In the present study, we verified the function of CaOx-Exo on autophagy and apoptosis of HK-2 cells *in vitro*. Caspase-3 is a key executor of apoptosis [33]. Bcl-2 protects many cells from apoptosis, and the main function of Bax is to promote cell apoptosis [34]. CaOx-Exo treatment enhanced Caspase-3 and Bax expression and repressed Bcl-2 expression in HK-2 cells, indicating that CaOx-exosomes induced apoptosis in HK-2 cells. Additionally, CaOx-Exo elevated the LC3-II/LC3-I ratio and Beclin-1 expression and decreased p62 expression in HK-2 cells. CaOx-Exo treatment also notably increased the GFP-LC3 puncta in HK-2 cells. The conversion of cytoplasmic LC3-I to autophagosome membrane-bound LC3-II suggests the occurrence of autophagy [35]. p62 is a receptor protein in the autophagy-lysosome pathway and a substrate protein of autophagy [36]. Therefore, CaOx-Exo activated autophagy in HK-2 cells. However, CaOx-Exo-induced cell apoptosis and autophagy were abrogated by 3-MA treatment. Thus, CaOx-Exo promoted apoptosis of HK-2 cells by promoting autophagy.

Obviously, there are some inadequacies in this work. This study initially revealed the impact of CaOx-treated macrophage-derived exosomes on apoptosis of HK-2 cells through *in vitro* experiments. Whether CaOx-treated macrophage-derived exosomes can affect kidney stone development *in vivo* still needs to be further explored. We will construct a CaOx stone mouse model by administration of glyoxylate and investigate the influence of CaOx-treated macrophage-exosomes on proximal tubular cell injury *in vivo*. Additionally, exosomes can carry DNA, miRNA and other biological molecules for information transmission. The signal molecules or signal pathways involved in the regulation of apoptosis and autophagy by exosomes need to be further studied.

## Conclusions

In conclusion, these data demonstrated that exosomes derived from CaOx-treated macrophages promoted apoptosis of HK-2 cells by promoting autophagy. Thus, this work suggests that CaOx-treated macrophages may plays a vital role in human proximal tubular cell apoptosis through exosome pathways.

## Highlights

- CaOx-macrophage-derived exosomes promoted apoptosis of HK-2 cells.
- CaOx-macrophage-derived exosomes promoted autophagy of HK-2 cells.
- Exosomes promoted apoptosis of HK-2 cells by promoting autophagy.
